# Melatonin Alleviates Intracerebral Hemorrhage-Induced Secondary Brain Injury in Rats via Suppressing Apoptosis, Inflammation, Oxidative Stress, DNA Damage, and Mitochondria Injury

**DOI:** 10.1007/s12975-017-0559-x

**Published:** 2017-08-01

**Authors:** Zhong Wang, Feng Zhou, Yang Dou, Xiaodi Tian, Chenglin Liu, Haiying Li, Haitao Shen, Gang Chen

**Affiliations:** Department of Neurosurgery & Brain and Nerve Research Laboratory, The First Affiliated Hospital of Soochow University, 188 Shizi Street, Suzhou, Jiangsu Province 215006 China

**Keywords:** Melatonin, Intracerebral hemorrhage, Secondary brain injury, Apoptosis, Inflammation, Oxidative stress, DNA damage, Mitochondria injury

## Abstract

Intracerebral hemorrhage (ICH) is a cerebrovascular disease with high mortality and morbidity, and the effective treatment is still lacking. We designed this study to investigate the therapeutic effects and mechanisms of melatonin on the secondary brain injury (SBI) after ICH. An in vivo ICH model was induced via autologous whole blood injection into the right basal ganglia in Sprague-Dawley (SD) rats. Primary rat cortical neurons were treated with oxygen hemoglobin (OxyHb) as an in vitro ICH model. The results of the in vivo study showed that melatonin alleviated severe brain edema and behavior disorders induced by ICH. Indicators of blood-brain barrier (BBB) integrity, DNA damage, inflammation, oxidative stress, apoptosis, and mitochondria damage showed a significant increase after ICH, while melatonin reduced their levels. Meanwhile, melatonin promoted further increasing of expression levels of antioxidant indicators induced by ICH. Microscopically, TUNEL and Nissl staining showed that melatonin reduced the numbers of ICH-induced apoptotic cells. Inflammation and DNA damage indicators exhibited an identical pattern compared to those above. Additionally, the in vitro study demonstrated that melatonin reduced the apoptotic neurons induced by OxyHb and protected the mitochondrial membrane potential. Collectively, our investigation showed that melatonin ameliorated ICH-induced SBI by impacting apoptosis, inflammation, oxidative stress, DNA damage, brain edema, and BBB damage and reducing mitochondrial membrane permeability transition pore opening, and melatonin may be a potential therapeutic agent of ICH.

## Introduction

Intracerebral hemorrhage (ICH) is a common acute nervous system disease with high mortality and disability, accounting for ~15% of all patients with stroke. Currently, an effective treatment modality for ICH is not available, with some patients resorting to hematoma evacuation, which is not satisfactory [1]. Seventy-five percent of patients that survive after sustaining ICH have varying degrees of motor, sensory, language, and other advanced neural function defects. While significant progress has been made to investigate the mechanisms of brain injury after ICH, an effective clinical treatment which can significantly improve ICH prognosis is still unavailable [1–5]. Previous studies indicated that ICH-induced brain injury is not only due to the hematoma mass effect and the potential hematoma expansion (they are the main causes of primary brain injury) but also due to secondary brain injury (SBI) [6]. Therefore, exploring how to reduce SBI and promote neural function recovery became the primary focus of researchers. The mechanisms contributing to SBI are very complex and mainly related to the following aspects: oxidative stress, neuronal death (including apoptosis and necrosis), inflammation, reactive oxygen species (ROS) generation, mitochondrial dysfunction, and so on. These mechanisms resulted in multiple pathological events in SBI, such as blood-brain barrier (BBB) integrity damage, brain edema, and brain injury. As many researches reported that these mechanisms are related to each other, but they have not been fully illustrated.

Melatonin (*N*-acetyl-5-methoxytryptamine) is a type of indolamine derived from tryptophan secreted mainly by the pineal gland, which has high biological availability and easily crosses the BBB to enter the brain parenchyma [7]. Recent studies showed that melatonin has high antioxidant properties: scavenging ROS; protecting mitochondrial oxidoreductase, superoxide dismutase (SOD), and other important proteins and enzymes which can alleviate DNA oxidative damage [8]; and reducing inflammation [9–14]. Additionally, previous research investigated the mechanisms of the protective effects of melatonin in brain injury caused by middle cerebral artery occlusion (MCAO) in a rat model of cerebral ischemia/reperfusion (I/R) [15]. Our previous study also showed that melatonin has beneficial effects against early brain injury after subarachnoid hemorrhage (SAH) in a rat model [16]; however, there are few reports that focus on the effects of melatonin in ICH. Therefore, the aim of this study was to explore the effects and mechanisms of melatonin on ICH-induced SBI.

## Methods

### Experimental Design, ICH Procedure, and Treatment Strategy

**Fig. 1:**
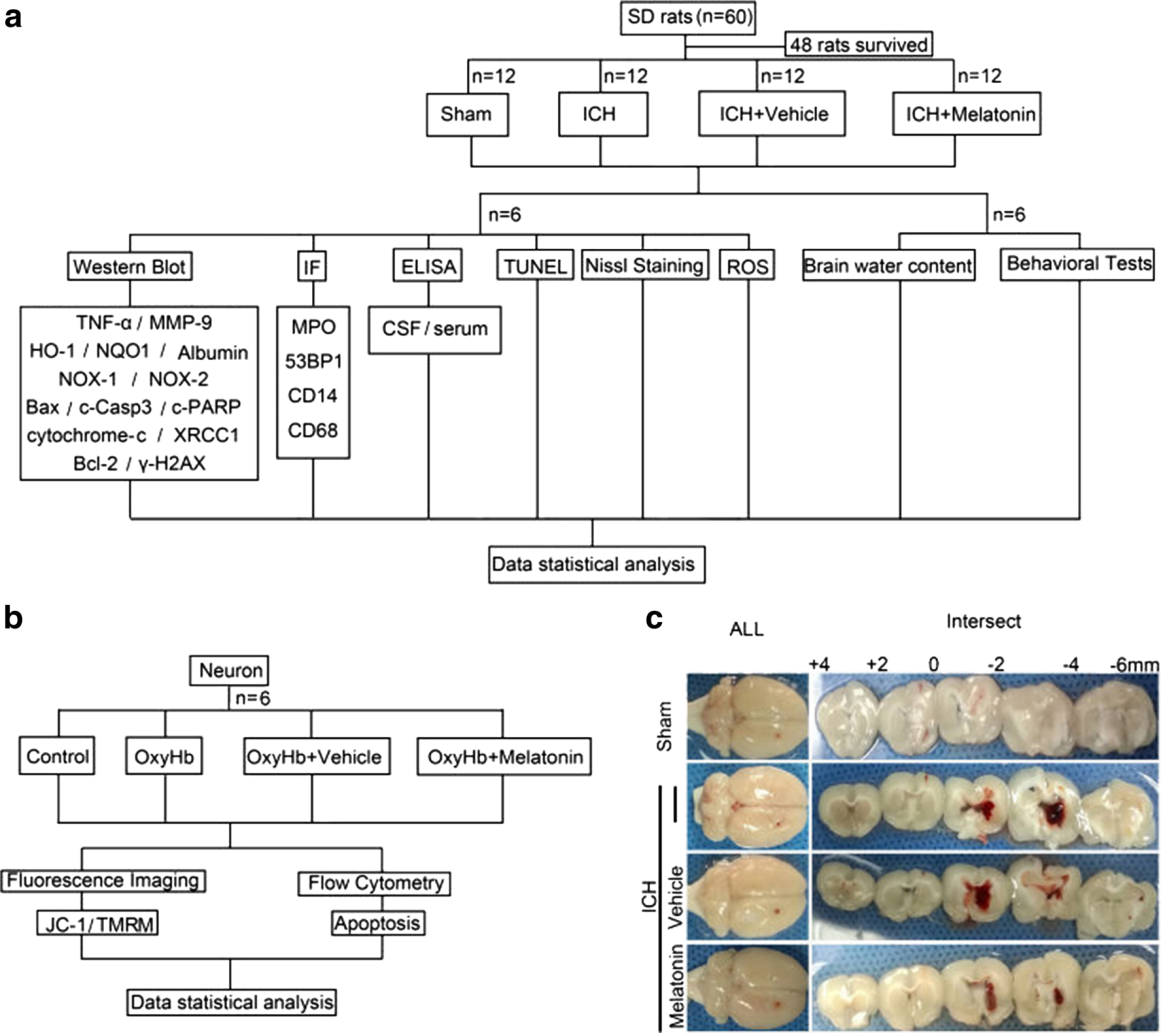
Experimental designs. **a** Experiment 1 was designed to investigate the effect of melatonin treatment on ICH-induced secondary brain injury (SBI) in vivo. **b** Experiment 2 was designed to investigate the effect of melatonin treatment on ICH-induced SBI in vitro. **c** Coronal sections of brain tissues after 72 h post-ICH induction

Adult male SD rats weighing 320–360 g (Animal Center of the Chinese Academy of Sciences, Shanghai, China) were randomly and equally divided into the following four groups: sham group, ICH group, ICH + vehicle group, and ICH + melatonin group (*n* = 12 in each group), using the table of random numbers by a technician who did not take part in this research. At 72 h after ICH, as before, all rats were examined for behavioral impairment, and then immediately, all rats were sacrificed, and blood and cerebrospinal fluid from each rat were collected. Six rats per group were sacrificed for western blot analysis, immunofluorescence analysis, TUNEL and Nissl staining, and ROS tests, and another six for brain edema in each group. For behavioral impairment and brain edema detection, the observers did not know the component of infusion or the group of rats. For western blot analysis, BBB permeability and ROS assay show quantitative results; each *n* represents data collected from one independent experiment using one rat; combined data from at least one independent experiment using six different rats are shown. For all the immunofluorescence analysis and TUNEL and Nissl staining, representative images from at least three independent experiments using six different rats are shown. The *n* is always defined as number of rats in every figure legend (Fig. 1).

The autologous whole blood model was utilized as previously described [17] due to its ability to closely simulate clinical ICH. SD rats were anesthetized intraperitoneally with 4% chloral hydrate, with additional chloral hydrate administered if needed based on tail pinch response. Once fully anesthetized, the specimens were set in a stereotactic apparatus frame (Shanghai Ruanlong Science and Technology Development Co., Ltd., China). Autologous whole blood (80 μl) was drawn by cardiac puncture and injected slowly (5 min) unilaterally with a microliter syringe into the right basal ganglia. The position of the basal ganglia was 3.5 mm lateral to the midline, 1.5 mm posterior to the bregma, and 5.5 mm ventral to the cortical surface. To prevent reflux, the needle stayed in place for an additional 5 min, and the scalp was then sutured. During the entire surgery, the rat was placed supine on a heating blanket to maintain body temperature between approximately 37 ± 0.5 °C [18, 19].

As described in previous studies [20], melatonin (Sigma, USA) was dissolved in absolute ethyl alcohol and diluted with 0.9% normal saline. A dose of melatonin (5 mg/kg), which was determined based on animal body weight, was injected intraperitoneally at 1, 24, and 48 h after ICH induction, with the animals sacrificed at 72 h after ICH. Vehicle-treated animals received an equal volume of the vehicle, which was also injected intraperitoneally.

### Isolation and Treatment of Primary Cortical Neuron

The procedure and protocol for primary cortical neuron isolation has been depicted in our previous report [21]. Briefly, cortical tissues were isolated from fetal SD rat brains at 18 days of gestation and treated with papain (100 mg/ml; Worthington, USA) for 10 min at 37 °C. Dissociated neurons were plated at a density of 20,000 cells/cm^2^ onto plates (Corning, USA) precoated with 0.1 mg/ml poly-d-lysine (Sigma, USA), cultured in Neurobasal medium supplemented with 2% B-27 and 0.5 mM GlutaMAX TM-I (all from GIBCO, USA), and maintained at 37 °C under humidified conditions and 5% CO_2_. Cells were maintained for 14–19 days, with half of the media exchanged for fresh media every 3 days. The neurons were then divided into four groups for the Annexin V and PI staining and mitochondrial membrane permeability transition pore (MPTP) assay as follows: control group; OxyHb (30 μM) treatment for 1 h; OxyHb + vehicle; and pretreatment with melatonin (60 μM) for 1 h, thorough rinsing, and OxyHb treatment for 1 h.

### ELISA

At 72 h post-ICH, blood and cerebrospinal fluid (CSF) were collected prior to sacrifice by puncturing the heart and foramen magnum. The CSF of all rats were immediately centrifuged for 30 min (4 °C, 12,000*g*), and the blood samples of all rats were centrifuged for 5 min (4 °C, 1000*g*); then, their supernatants were collected to measure TNF-α and IL-1β levels using specific ELISA kit (Bio-Swamp, China) according to the manufacturers’ instructions.

### ROS Assay

The levels of ROS in brain tissues were detected by the Reactive Oxygen Species Assay Kit (Beyotime, China). The collected tissues were first homogenized and centrifuged at 12,000*g* for 10 min at 4 °C. The supernatants were used for the ROS assay. ROS concentrations were evaluated using the oxidant-sensitive probe 2,7-dichlorofluorescein diacetate (DCF-DA) according to the manufacturer’s instructions. A fluorometric microplate reader (FilterMax F5, Molecular Devices, Sunnyvale, USA) with excitation and emission at 485 and 530 nm, respectively, was used to measure the fluorescence intensity, and the samples of all rats were detected in at least one dependent experiment. The concentrations of ROS were expressed as fluorescence intensity per milligram protein, and the results of all groups were normalized to the sham group and served as the relative levels of oxidative stress.

### Brain Water Content and Behavioral Tests

As described in a previous study, at 72 h post-ICH induction, rats were injected intraperitoneally with 4% chloral hydrate, with the intact brain tissues removed immediately [20]. The brain tissues were divided into two hemispheres along the midline, with each hemisphere divided into two parts containing the cortex and basal ganglia. The obtained samples and the cerebellum were then divided into the following five groups: contralateral cortex (Cont-CX), contralateral basal ganglia (Cont-BG), ipsilateral cortex (Ipsi-CX), ipsilateral basal ganglia (Ipsi-BG), and cerebellum (CB; all groups: *n* = 6). The brain tissues were immediately weighed with an electronic analytical balance and the wet weight was recorded. The brain tissues were then dried in an electric thermostatic drier at 100 ± 5 °C for 72 h until the sample weights were consistent to obtain the dry weight and calculated as follows: water content of brain tissues = (wet weight − dry weight) / (wet weight) × 100%.

Behavioral testing was performed at 1 h before sacrifice. All the rats in each group were examined using a previously published scoring system and monitored for appetite, activity, and neurological defects (details shown in Table 1) [22].

**Table 1:** Neurobehavioral evaluation

Category	Behavior	Score
Appetite	Finished meal	0
	Left meal unfinished	1
	Scarcely ate	2
Activity	Walk and reach at least three corners of the cage	0
	Walk with some stimulations	1
	Almost always lying down	2
Deficits	No deficits	0
	Unstable walk	1
	Impossible to walk	2

### BBB Injury

BBB permeability was assessed on the basis of albumin extravasation [23]. Generally, albumin concentration in the brain is very low because of the existence of BBB, but the content of albumin in brain tissues increases obviously once BBB is damaged. Therefore, the changes of albumin concentration can serve as an indicator to estimate the degree of BBB injury [24, 25]. The western blot analysis was used to test the protein levels of albumin in brain tissues of rats in each group.

### Nissl Staining

After coronal sections had been deparaffinized and rehydrated, the slides were stained in toluidine blue solution for 40 min at 50–60 °C. After clearing in distilled water, the slides were gradually dehydrated for 3 min in successive baths of ethanol, with one pass each in 70, 80, and 95% and two passes in 100%. All slides were then given two 5 min passes in 100% dimethylbenzene and coverslips were applied with neutral balsam. Finally, the numbers of surviving neurons per ×400 field within the hippocampal CA1 were counted.

### TUNEL Staining

TUNEL staining was utilized to detect cellular apoptosis in the brain tissues around the hematoma from all of the groups according to the manufacturer’s protocol (In Situ Cell Death Detection Kit, Roche, Germany). The TUNEL-positive cells in the brain tissues around the hematoma were observed and analyzed using a fluorescence microscope (Olympus Co., Japan).

### Western Blot Analysis

Western blot analysis was performed as described previously [26]. Briefly, brain samples around the hematoma were collected, homogenized, and lysed separately in ice-cold RIPA lysis buffer (Beyotime, China). The samples were then centrifuged for 10 min (4 °C, 12,000*g*). The supernatants were collected immediately and the protein concentrations were determined using a bicinchoninic acid (BCA) kit (Beyotime, China) according to the manufacturer’s instructions. The protein samples (60 μg/lane) were separated by 10 or 12% SDS polyacrylamide gel and electrotransferred to nitrocellulose filter membranes (Millipore, USA). The membranes were blocked with 5% skim milk for 1 h at room temperature and then incubated with primary antibodies overnight at 4 °C. The membranes were then washed with TBST and incubated with horseradish peroxidase (HRP)-conjugated secondary antibody for 2 h at room temperature. The protein bands were visualized using enhanced chemiluminescence (ECL), and the relative protein quantity was determined using ImageJ software (National Institutes of Health, USA).

For the release of cytochrome c into the cytoplasm, we isolated the mitochondria from the protein extraction of the brain tissues by Cell Mitochondria Isolation Kit (Beyotime, China), and then western blot analysis was used to measure cytochrome c in residual protein sample.

The primary antibodies used include the following: matrix metalloproteinase (MMP)-9 (Abcam, USA), NADPH oxidase (NOX)-1 (Santa Cruz, USA), NOX-2 (Abcam, USA), heme oxygenase (HO)-1 (Santa Cruz, USA), NAD(P)H quinone oxidoreductase (NQO) 1 (Santa Cruz, USA), Bcl-2 (Abcam, USA), BAX (Abcam, USA), cleaved caspase 3 (Abcam, USA), cleaved poly(ADP-ribose) polymerase (c-PARP) (Abcam, USA), X-ray repair complementing defective repair in Chinese hamster cells (XRCC) 1 (Santa Cruz, USA), gamma histone H2AX (γ-H2AX) (Abcam, USA), cytochrome c (Santa Cruz, USA), and β-tubulin (Santa Cruz, USA) as a loading control. HRP-conjugated anti-IgG (Santa Cruz, USA) was used as secondary antibodies.

### Immunofluorescence (IF) Staining

The brain tissues was embedded in paraffin and sectioned at 4 μm, and immunofluorescence staining for myeloperoxidase (MPO), p53-binding proteins (53BP) 1, CD14, and CD68 was performed. The sections were incubated with primary MPO, 53BP1, CD14, and CD68 (all diluted in 1:100; Santa Cruz, USA) antibodies overnight at 4 °C. Secondary antibody (Life Technologies, USA, 1:300 dilution) was added and incubated for 1 h at 37 °C, and the sections were then washed three times with PBST. After final washing, sections were protected with coverslips, with the nucleus visualized with DAPI (Southern Biotech, USA). The brain tissues around the hematoma were observed and analyzed using a fluorescence microscope (Olympus Co., Japan), and the relative intensities were determined using ImageJ software (National Institutes of Health, USA).

### Annexin V and PI Staining In Vitro

After various treatments, neurons were trypsinized with 0.25% trypsin (without EDTA) and centrifuged at 300*g* for 5 min, and the cell pellet was resuspended in 500 μl binding buffer, with 5 μl Annexin V and 5 μl PI (Beyotime, China) added. After incubation for 20 min in the dark at 37 °C, the cells were analyzed by flow cytometry (FACS Calibur, BD, USA) and at least 20,000 events per sample were recorded.

### JC-1 and TMRM Staining In Vitro

Tetrechloro-tetraethylbenzimidazol carbocyanine iodide (JC-1) and tetramethylrhodamine methyl ester perchlorate (TMRM) staining were used to detect neuron MPTP opening according to the manufacturer’s protocol (Mitochondrial membrane potential assay kit with JC-1, Beyotime, China; TMRM, Santa Cruz, USA) [27–29]. Pretreated neurons were washed three times with PBS, followed by the additional 1 ml JC-1 working solution per sample and incubated at 37 °C for 20 min. After incubation, the neurons were washed twice with JC-1 staining buffer and coverslipped with neutral balsam.

TMRM was dissolved in DMSO and diluted with PBS, with 1 ml TMRM solution added to each sample and incubated at 37 °C for 20 min. After incubation, the neurons were washed three times with PBS and coverslipped with neutral balsam. These results were visualized using a fluorescence microscope (Olympus Co., Japan), and the relative fluorescence intensities of JC-1 and TMRM were determined using ImageJ software (National Institutes of Health, USA).

### Statistical Analysis

All data were expressed as mean ± SEM and GraphPad Prism 6.0 was adopted for all statistical analyses. Data sets were tested for normality of distribution with Kolmogorov-Smirnov test. Data groups (two groups) with normal distribution were compared using two-sided unpaired Student’s *t* test, and the Mann-Whitney *U* test was used for nonparametric data. *P* < 0.05 was considered as statistically significant difference.

## Results

### Melatonin Attenuated Neurological Behavior Impairment, BBB Disruption, and Brain Water Content in Brain Tissues After ICH

**Fig. 2:**
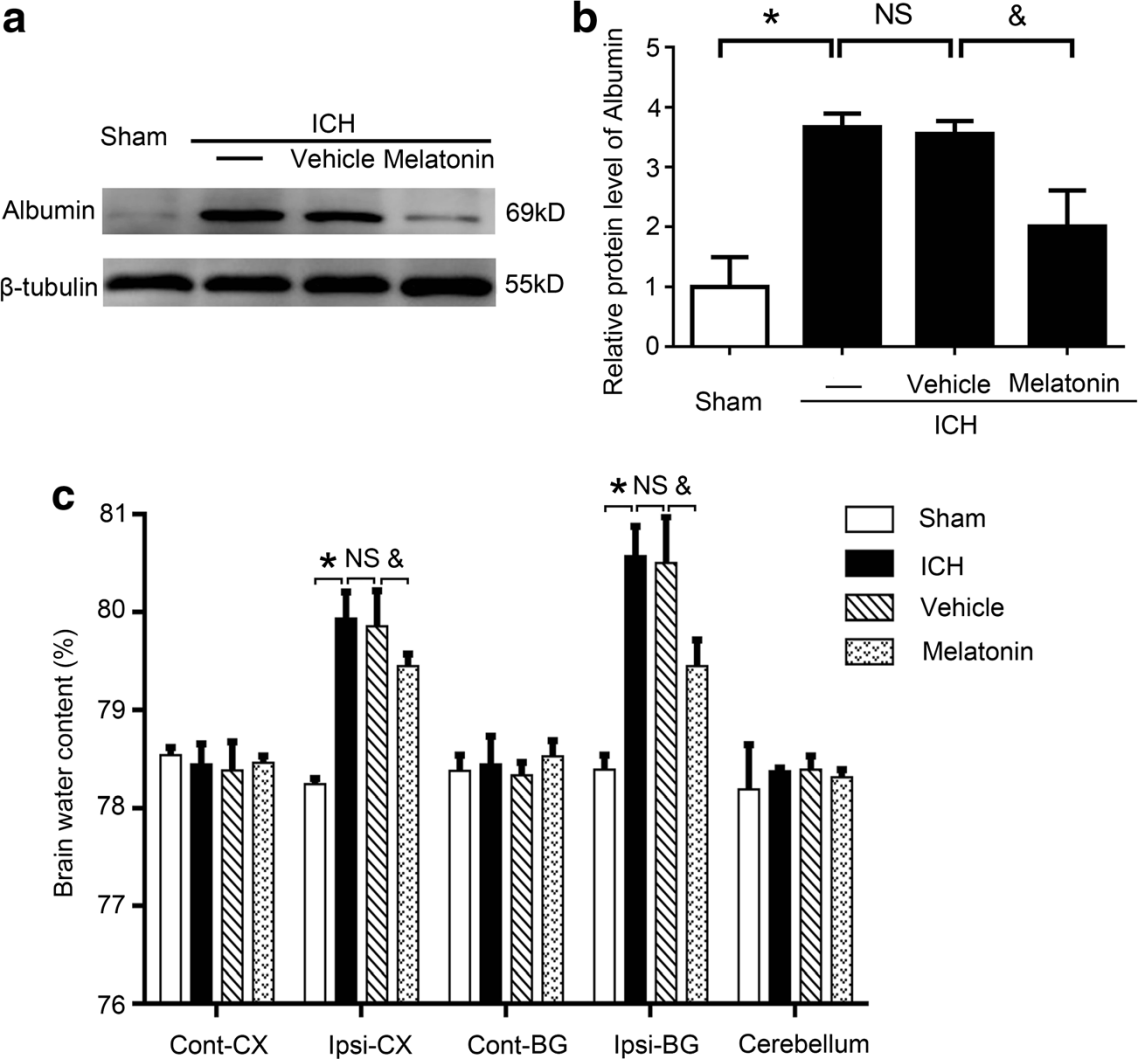
Evaluation of blood-brain barrier (BBB) disruption and brain water content of brain tissues at 72 h post-ICH induction. **a** Western blot analysis examines the albumin level of the sham, ICH, ICH + vehicle, and ICH + melatonin groups. **b** Relative albumin levels were calculated based on densitometry analysis. The mean albumin level of the sham group was normalized to 1.0. **c** Recorded brain water content at 72 h post-ICH. All data are displayed as means ± SEM, with *P < 0.05 and ^&^ *P* < 0.05 deemed as significant difference; *NS*, no significant difference, *n* = 6

To define the effects of melatonin in neurological behavioral impairment after ICH, we performed behavioral testing at 1 h before sacrifice. At 72 h after ICH induction, the rats showed severe neurological behavioral impairment compared to the sham group. After receiving an intraperitoneal melatonin injection, the impairment was ameliorated (Table 2). We further evaluated the levels of albumin in each group, which is an important hallmark of BBB disruption. In the ICH group, albumin levels in the brain tissues were significantly increased when compared to the sham group, while melatonin treatment significantly reduced the albumin levels induced by ICH (Fig. 2a, b).

**Table 2:** Clinical behavioral scores in each group

Group (*n* = 12)	Scores (mean ± SEM)
Sham	0.333 ± 0.211
ICH	3.167 ± 0.307*
ICH + vehicle	3.500 ± 0.224 ns
ICH + melatonin	2.500 ± 0.342^#^

*P < 0.05 vs. sham group; *ns*, no significant difference vs. ICH group; #*P* < 0.05 vs. ICH + vehicle group

Additionally, brain water content was examined to explore the effects of melatonin treatment on ICH-induced brain edema. No significant differences were noted in the contralateral cortex, contralateral basal ganglia, or cerebellum in these four groups (Fig. 2c). However, in the ipsilateral cortex and ipsilateral basal ganglia of these four groups, the ICH group showed significant increases in water content compared to the sham group, while melatonin treatment significantly impaired the brain water content. These results indicated that melatonin treatment is able to ameliorate brain injury (including neurological behavioral impairment, BBB disruption, and brain edema) after ICH.

### Melatonin Inhibited Oxidative Stress in Brain Tissues at 72 h After ICH

**Fig. 3:**
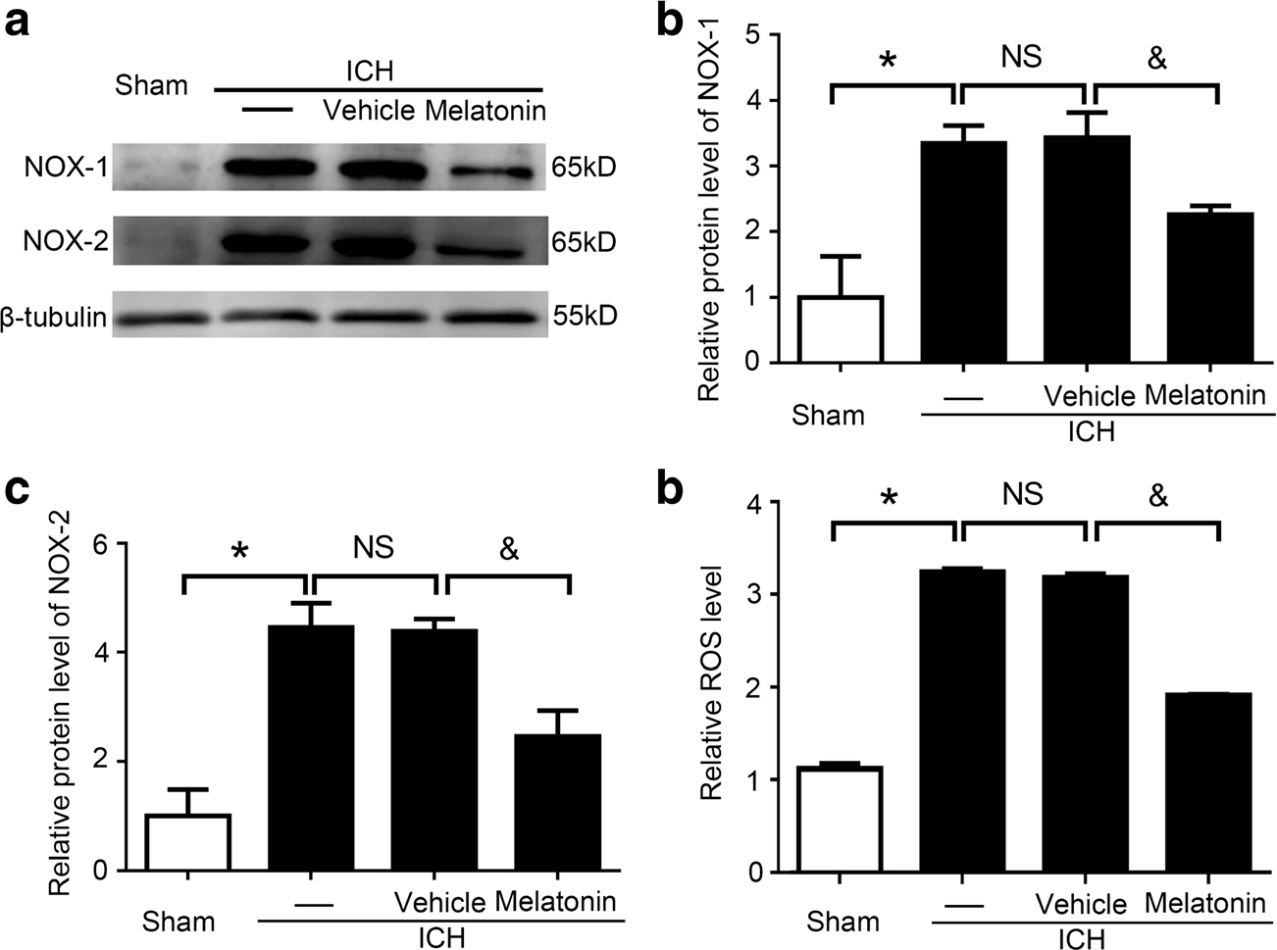
Oxidative stress indicator expression levels in brain tissues at 72 h after ICH. **a** Western blot analysis shows protein levels of NOX-1 and NOX-2 in the sham, ICH, ICH + vehicle, and ICH + melatonin groups. **b**, **c** Relative NOX-1 and NOX-2 expression level calculations based on densitometry analysis. The mean values of NOX-1 and NOX-2 within the sham group were normalized to 1.0. All data are displayed as a mean ± SEM, with *P < 0.05 and ^&^ *P* < 0.05 deemed significant difference. *NS*, no significant difference compared to the sham group (*n* = 6). **d** Effects of melatonin treatment on brain ROS levels at 72 h post-ICH. The mean ROS value of the sham group was normalized to 1.0. All data are displayed as a mean ± SEM, with *P < 0.05 and ^&^ *P* < 0.05 deemed as significant difference. *NS*, no significant difference compared to the sham group (*n* = 6)

To explore the effects of melatonin in oxidative stress after ICH, the protein levels of NOX-1 and NOX-2, which are indicators of oxidative stress, were detected by western blot analysis at 72 h after ICH. The results showed that these two indicators were significantly increased in the ICH group compared to the sham group, while no significant difference between the ICH and ICH + vehicle groups was noted. However, melatonin treatment significantly decreased expression levels of these two indicators when compared to the ICH + vehicle group (Fig. 3a–c).

Additionally, the ROS levels in these four groups were also measured at 72 h after ICH induction (Fig. 3d). The results showed that the ROS level was significantly increased in the ICH group compared to the sham group, while melatonin treatment remarkably reduced ROS level in brain tissues. These results suggested that melatonin can inhibit oxidative stress in brain tissues after ICH.

### Melatonin Impaired Inflammation in Brain Tissues at 72 h Post-ICH Induction

**Fig. 4:**
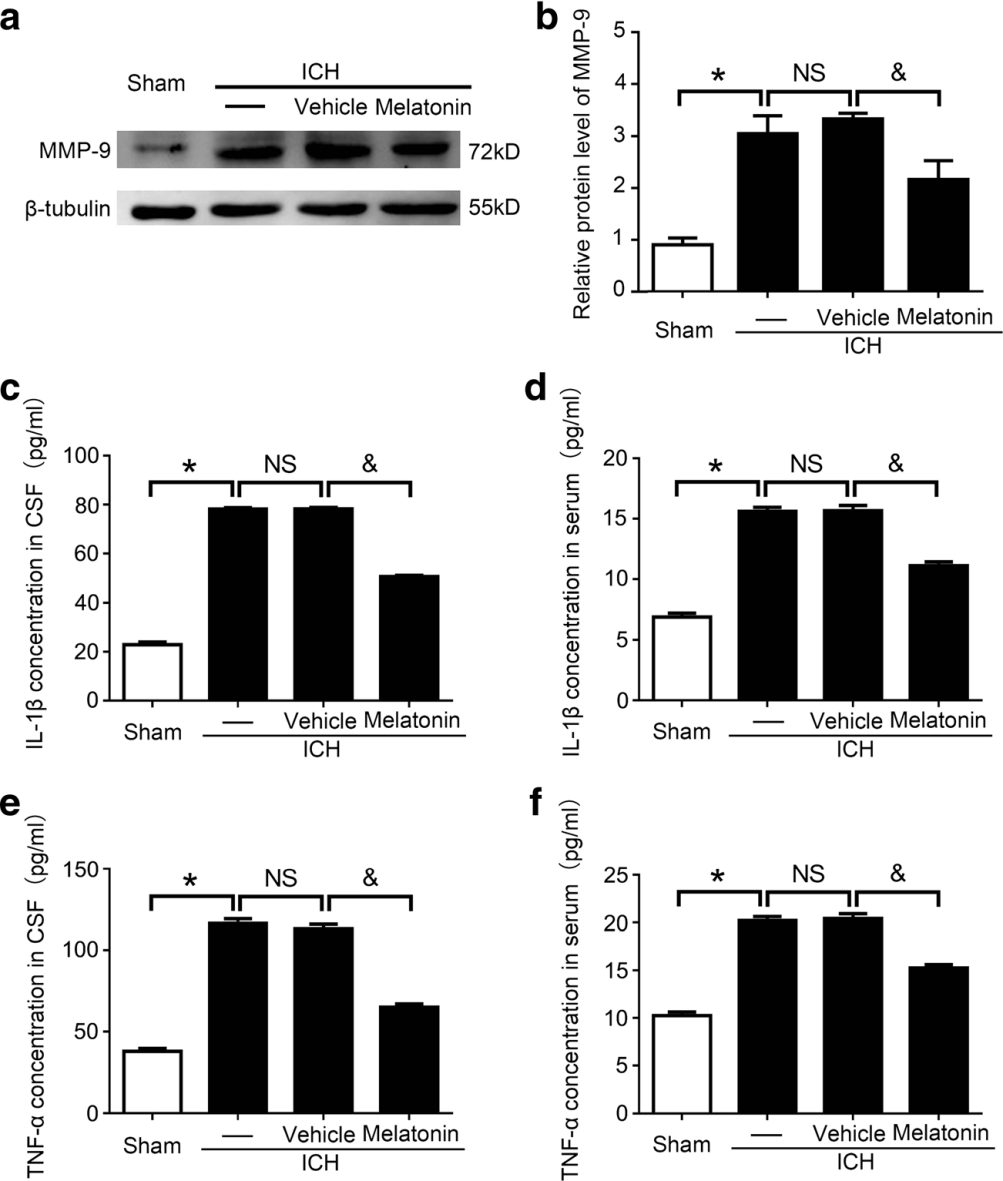
Inflammatory cytokines expression levels in brain tissues at 72 h post-ICH. **a** Western blot analysis examines MMP-9 in the sham, ICH, ICH + vehicle, and ICH + melatonin groups. **b** Relative MMP-9 expression level calculation based on densitometry analysis. The mean values of MMP-9 in the sham group were normalized to 1.0. All data are displayed as mean ± SEM, with *P < 0.05 and ^&^ *P* < 0.05 deemed as significant difference; *NS*, no significant difference, *n* = 6. **c**–**f** TNF-α and IL-1β levels in the CSF and serum at 72 h post-ICH. All data are displayed as mean ± SEM, with *P < 0.05 and ^&^ *P* < 0.05 deemed as significant difference; *NS*, no significant difference, *n* = 6

To determine whether melatonin inhibits inflammation after ICH, western blot analysis was used to measure the protein level of MMP-9 in brain tissues, which is an indicator of inflammation. The results showed that, compared to the sham group, the level of MMP-9 was significantly increased in the ICH group, while melatonin treatment obviously decreased its level compared to the ICH + vehicle group (Fig. 4a, b).

Furthermore, the levels of the pro-inflammatory cytokines (IL-1β and TNF-α) in the CSF and serum from rats in these four groups were elevated by ELISA. The results confirmed that IL-1β and TNF-α levels were significantly increased in the ICH group compared to the sham group. However, melatonin treatment significantly decreased the levels of these two cytokines compared to the ICH + vehicle groups (Fig. 4c–f).

**Fig. 5:**
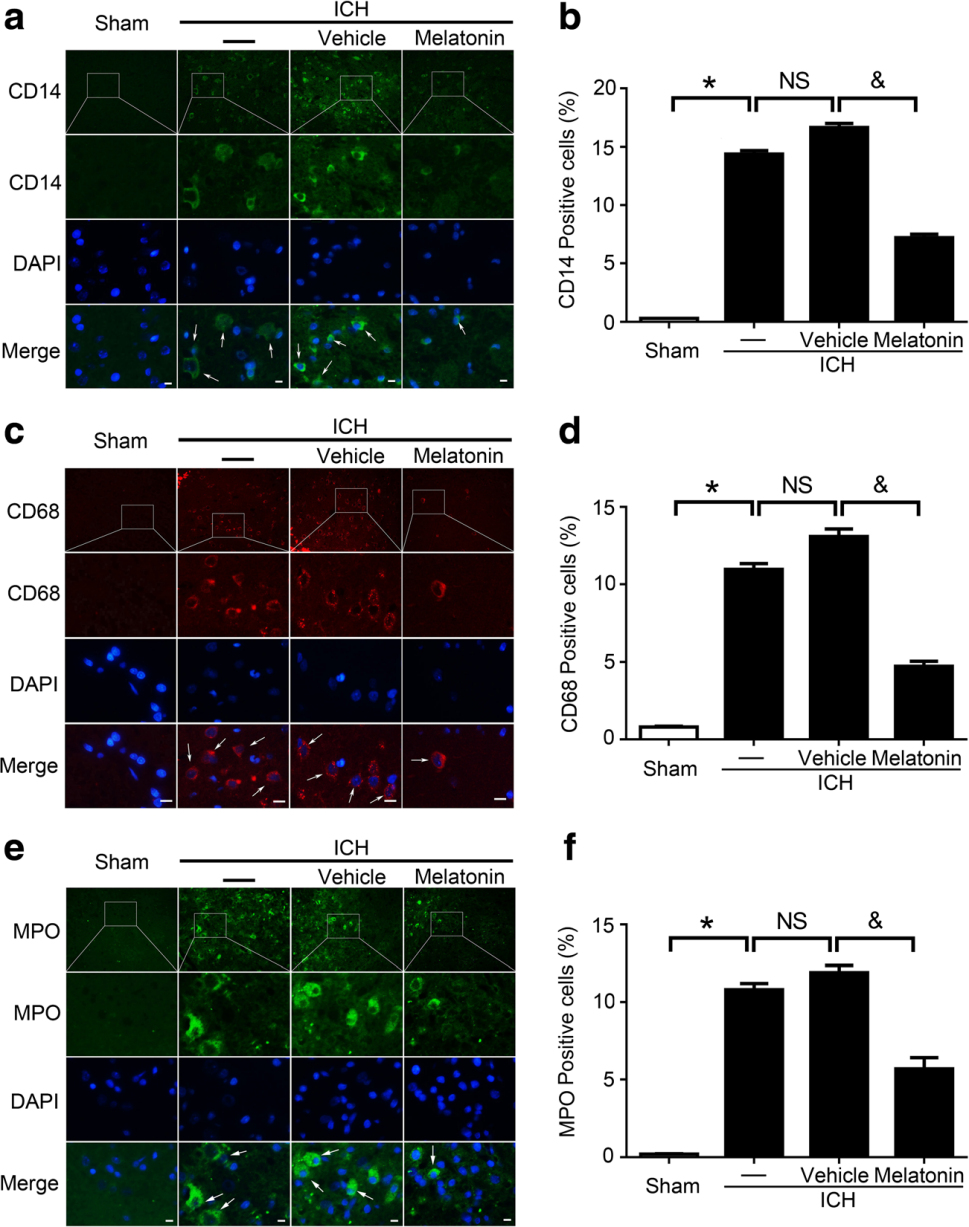
Immunofluorescence (IF) staining for the identification of inflammatory cells around the hematoma in brain tissues at 72 h post-ICH. Representative IF staining to identify CD14-positive (**a**), CD68-positive (**c**), and MPO-positive (**e**) cells (*green* or *red*), with the nuclei fluorescently labeled with 4,6-diamino-2-phenyl indole (*DAPI*, *blue*); *scale bar* = 32 μm. Percentage of CD14-positive (**b**), CD68-positive (**d**), and MPO-positive (**f**) cells around the hematoma in the brain tissues. *Arrows* indicate CD14-positive, CD68-positive, and MPO-positive cells. All data are displayed as mean ± SEM, with *P < 0.05 and ^&^ *P* < 0.05 deemed as significant difference; *NS*, no significant difference, *n* = 6

To explore infiltration of inflammatory cells in the brain tissues around the hematoma after ICH, the indicators (CD14, CD68, and MPO) of the inflammatory cells were examined by immunofluorescence staining. The results showed that, relative to the sham group, the positive ratios of these indicators were increased in the ICH groups. Nevertheless, these positive ratios were reduced significantly following melatonin treatment (Fig. 5a–f). These findings suggested that melatonin treatment can inhibit inflammation in brain tissues after ICH.

### Melatonin Reduced DNA Damage in Brain Tissues at 72 h Post-ICH Induction

**Fig. 6:**
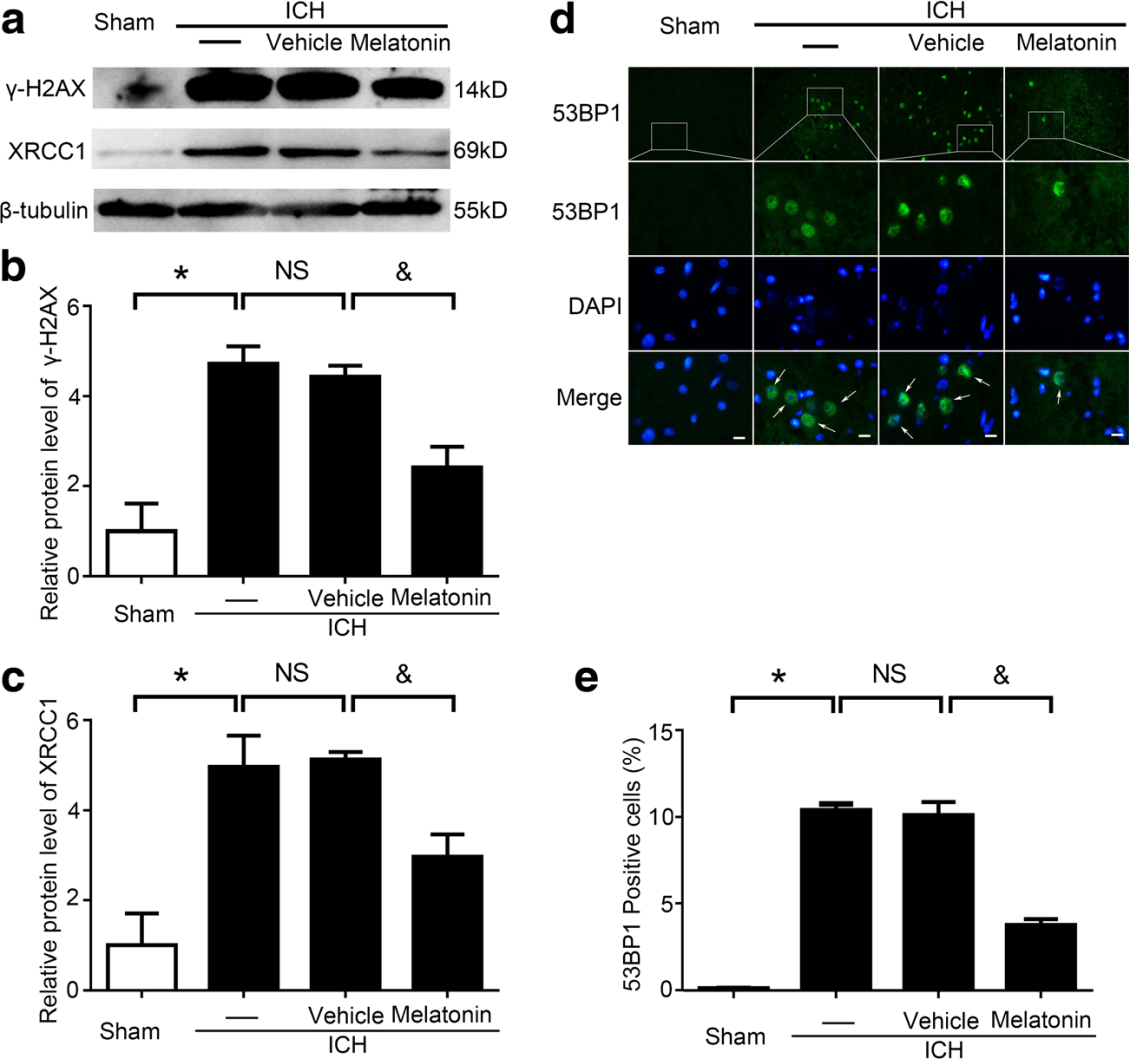
DNA damage indicator expression and immunofluorescence (IF) staining to visualize DNA-damaged cells around the hematoma in brain tissues 72 h post-ICH induction. **a** Western blot analysis examining XRCC1 and γ-H2AX levels in the sham, ICH, ICH + vehicle, and ICH + melatonin groups. **b**, **c** Relative XRCC1 and γ-H2AX levels were calculated based on densitometry analysis. The mean values of XRCC1 and γ-H2AX within the sham group were normalized to 1.0. All data are displayed as mean ± SEM, with *P < 0.05 and ^&^ *P* < 0.05 deemed as significant difference; *NS*, no significant difference, *n* = 6. **d** Representative IF staining to identify 53BP1-positive cells (*green*) and with nuclei fluorescently labeled with 4,6-diamino-2-phenyl indole (*DAPI*, *blue*). *Arrows* indicate 53BP1-positive cells; *scale bar* = 32 μm. **e** Percentage of 53BP1-positive cells around the hematoma in the brain. All data are displayed as mean ± SEM, with *P < 0.05 and ^&^ *P* < 0.05 deemed as significant difference; *NS*, no significant difference, *n* = 6

The protein levels of γ-H2AX and XRCC1, two indicators of DNA damage, were detected by western blot analysis, and the results showed that, compared to the sham group, they were obviously increased in the ICH group, while no significant difference was noted between the ICH and ICH + vehicle groups. Furthermore, the levels of γ-H2AX and XRCC1 were significantly reduced by melatonin treatment when compared to the ICH + vehicle group (Fig. 6a–c).

53BP1 is also an indicator of DNA damage. The numbers of 53BP1-positive cells in brain tissues around the hematoma were increased significantly in the ICH group compared to the sham group, and no significant difference was noted between the ICH and ICH + vehicle groups. However, the ratio of 53BP1-positive cells was reduced remarkably in the ICH + melatonin group compared to the ICH + vehicle group (Fig. 6d, e). These results suggested that melatonin can alleviate ICH-induced DNA damage in brain tissues.

### Melatonin Promoted Antioxidant in Brain Tissues at 72 h After ICH

**Fig. 7:**
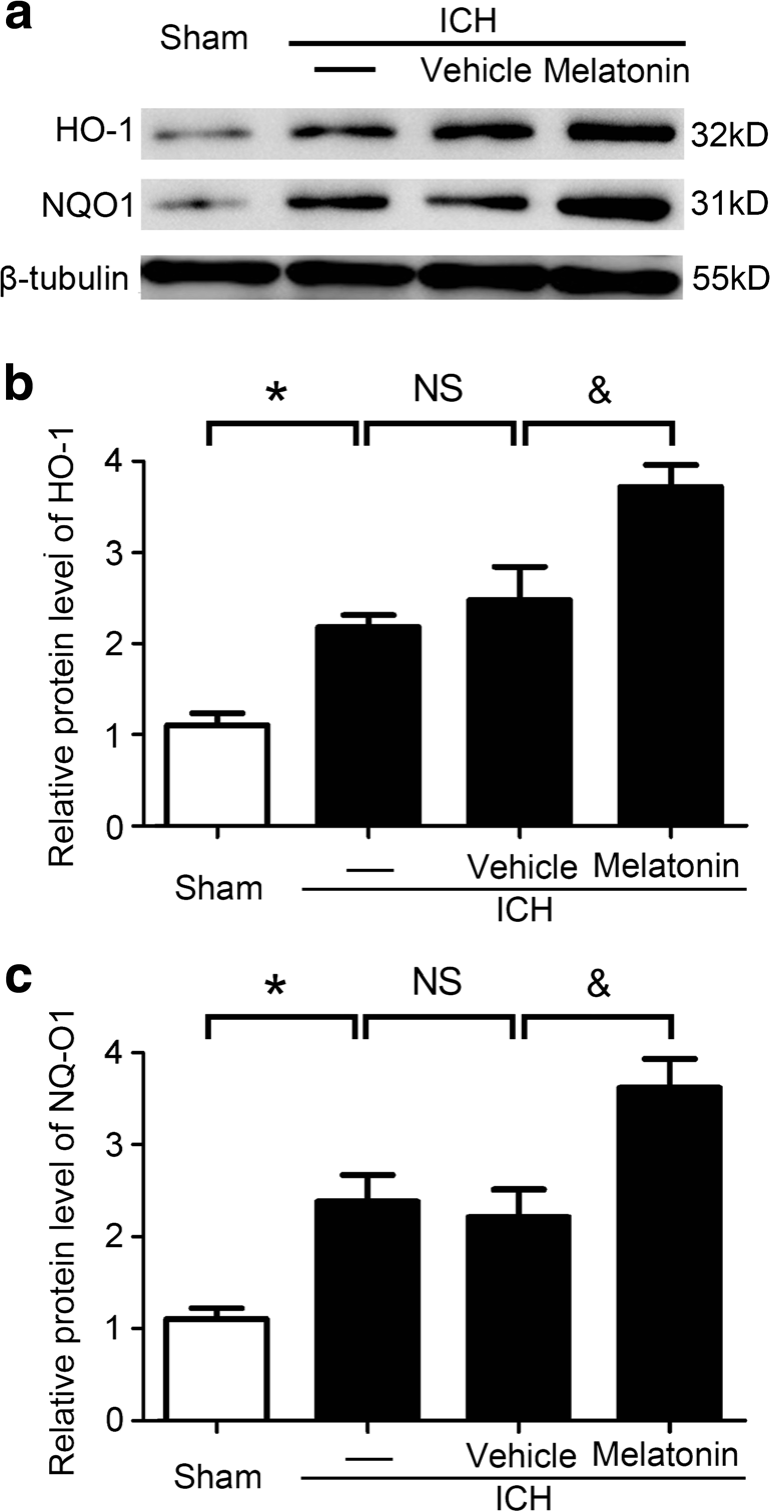
Melatonin increased antioxidant indicator expressions in brain tissues at 72 h after ICH. **a** Western blot analysis displayed the expressions of HO-1 and NQO1 in the sham, ICH, ICH + vehicle, and ICH + melatonin groups. Relative HO-1 (**b**) and NQO1 (**c**) levels were calculated based on densitometry analysis. The mean values of the HO-1 and NQO1 within the sham group were normalized to 1.0. All data are displayed as mean ± SEM, with *P < 0.05 and ^&^ *P* < 0.05 deemed as significant difference; *NS*, no significant difference, *n* = 6

The results of western blot analysis demonstrated that, compared with the sham group, the protein levels of HO-1 and NQO1, two indicators of antioxidant, were significantly increased in the ICH group, while there was no significant difference between the ICH and ICH + vehicle groups. Meanwhile, melatonin treatment increased HO-1 and NQO1 levels compared to the ICH + vehicle group (Fig. 7a–c). The results suggested that melatonin plays an important role in alleviating oxidative stress by promoting antioxidant in brain tissues after ICH.

### Neuroprotective Effects of Melatonin Against the Neuron Death Induced by ICH

**Fig. 8:**
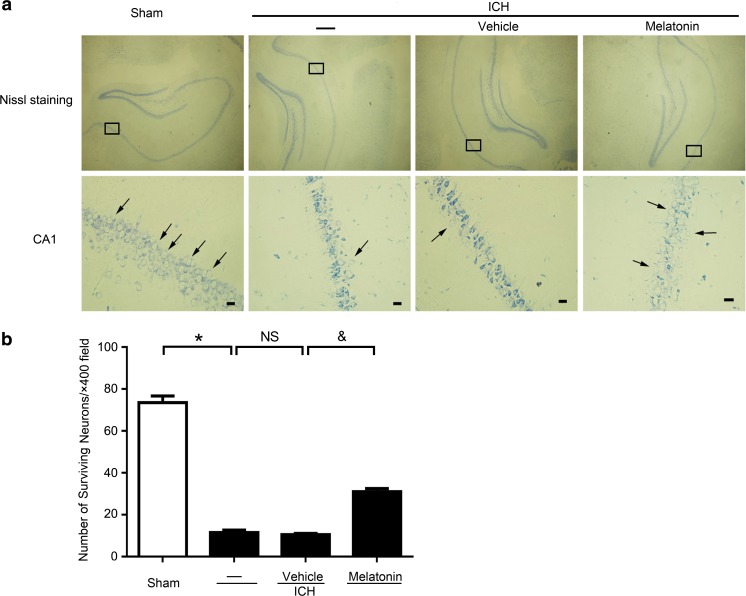
Neuroprotective effects of melatonin against ICH-induced neuronal death. **a** Representative Nissl staining sections of the hippocampus and hippocampal CA1 region in rats. *Arrows* indicate surviving neurons; *scale bar* = 32 μm. **B** quantitative analysis of the numbers of surviving neurons per ×400 field in the hippocampal CA1 region. All data are displayed as mean ± SEM, with *P < 0.05 and ^&^ *P* < 0.05 deemed as significant difference; *NS*, no significant difference, *n* = 6

We used Nissl staining to further assess whether melatonin has the protective effects on neurons in the CA1 region of the hippocampus after ICH. The sham group hardly had any neuronal death. The numbers of surviving neurons in the ICH group were significantly reduced to that in the sham group, but melatonin treatment remarkably increased it. There was no significant difference between the ICH and ICH + vehicle groups (Fig. 8a, b). These results showed that melatonin was against neuron death in brain tissues after ICH.

### Melatonin Inhibited Apoptosis in Brain Tissues After ICH Both In Vivo and In Vitro

**Fig. 9:**
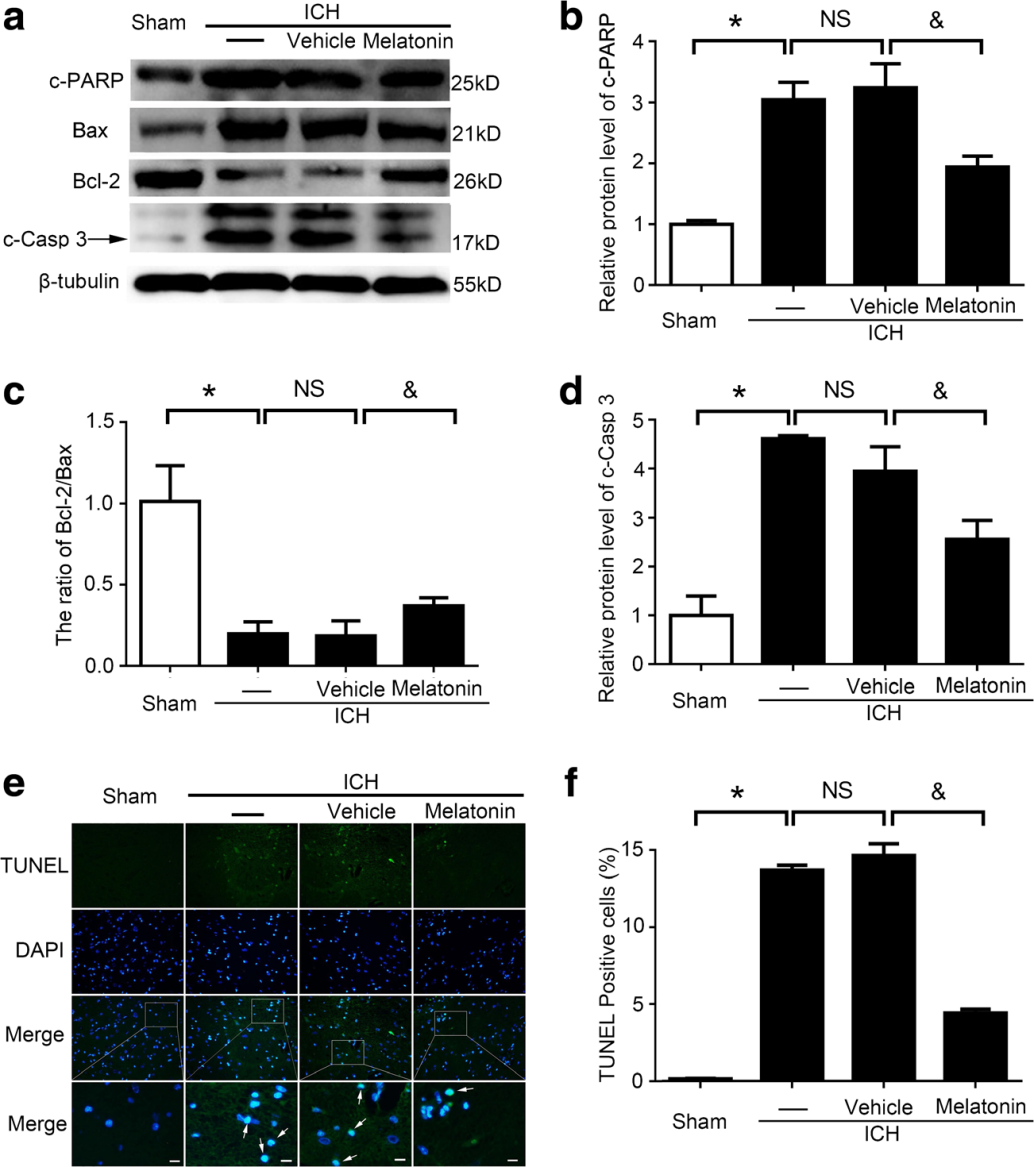
Melatonin inhibited apoptosis in brain tissues at 72 h after ICH. **a** Western blot analysis shows c-PARP, Bcl-2, Bax, and cleaved caspase 3 levels in the sham, ICH, ICH + vehicle, and ICH + melatonin groups. **b**–**d** Relative c-PARP, cleaved caspase 3 levels, and the ratio of Bcl-2/Bax were calculated based on densitometry analysis. The mean values of c-PARP, cleaved caspase 3, and the ratio of Bcl-2/Bax in the sham group were normalized to 1.0. All data are displayed as mean ± SEM, with *P < 0.05 and ^&^ *P* < 0.05 deemed as significant difference; *NS*, no significant difference, *n* = 6. **e** Double staining for terminal deoxynucleotidyl transferase-mediated dUTP nick-end labeling (*TUNEL*, green) and 4,6-diamino-2-phenyl indole (*DAPI*, *blue*). *Arrows* indicate TUNEL-positive cells; *scale bar* = 32 μm. **f** Percentage of TUNEL-positive cells around the hematoma in the brain tissues. All data are displayed as mean ± SEM, with *P < 0.05 and ^&^ *P* < 0.05 deemed as significant difference; *NS*, no significant difference, *n* = 6

To explore the effects of melatonin in cell apoptosis after ICH, the expression levels of cleaved caspase 3 and cleaved PARP, two indicators of apoptosis, were tested by western blot analysis. The results suggested that, compared to the sham group, there was a significant increase in the ICH group, while this upregulation was obviously decreased by melatonin treatment. Another indicator of apoptosis, the ratio of Bcl-2/BAX, was decreased in the ICH group compared to the sham group, but melatonin treatment increased the ratio of Bcl-2/BAX in brain tissues after ICH (Fig. 9a–d). Microscopically, TUNEL staining was utilized to explore the effects of melatonin treatment on apoptosis in the brain tissues at 72 h post-ICH induction. The numbers of TUNEL-positive cells in the brain tissues around the hematomas were significantly increased in the ICH groups compared to the sham group, while a reduction was noted in the ICH + melatonin group (Fig. 9e, f).

**Fig. 10:**
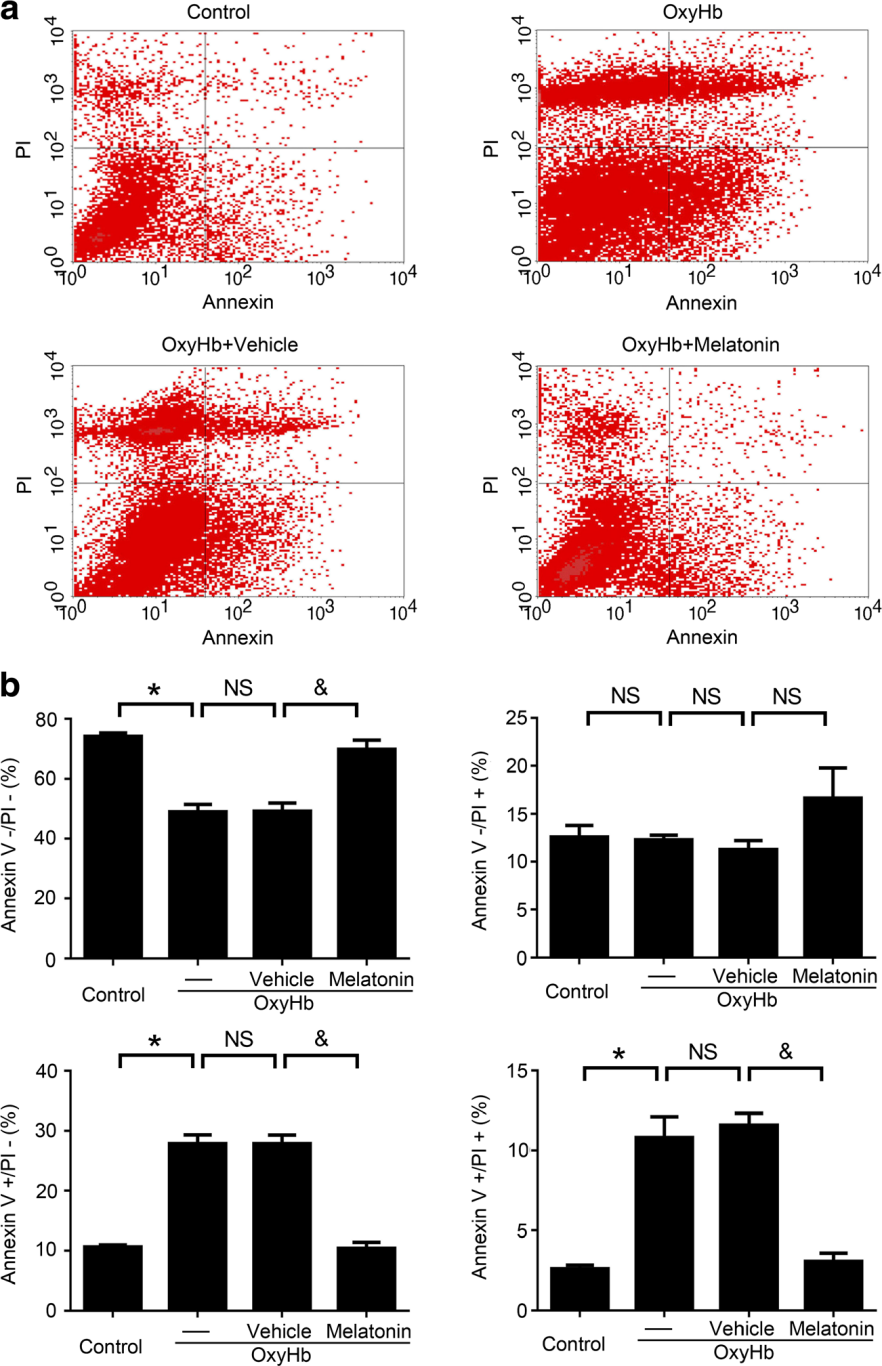
Melatonin inhibited OxyHb-induced neuronal apoptosis. **a** Neuronal apoptosis in various groups were detected via PI and Annexin V double staining and flow cytometry analysis in vitro. **b** *Bar graphs* showing different conditions of neurons in various groups: PI−/Annexin V− represents survival neurons, PI+/Annexin V− represents necroptotic neurons, PI−/Annexin V+ represents apoptotic neurons, and PI+/Annexin V+ represents neurons with mixed damage. All data are displayed as mean ± SEM, with *P < 0.05 and ^&^ *P* < 0.05 considered significant; *NS*, no significant difference, *n* = 6

In the in vitro experiments, after various treatments, neurons were digested to generate cell suspension, stained with Annexin V and PI, and examined by flow cytometry. The results showed that the apoptotic ratio was higher in the OxyHb treatment group relative to the control group. Additionally, the apoptotic ratio of neurons in the OxyHb + melatonin group was significantly decreased compared to the OxyHb + vehicle group (Fig. 10a, b). These results indicated that melatonin can inhibit apoptosis in neurons induced by ICH both in vivo and in vitro.

### Melatonin Suppressed the MPTP Opening and Mitochondrial Damage After ICH Both In Vivo and In Vitro

**Fig. 11:**
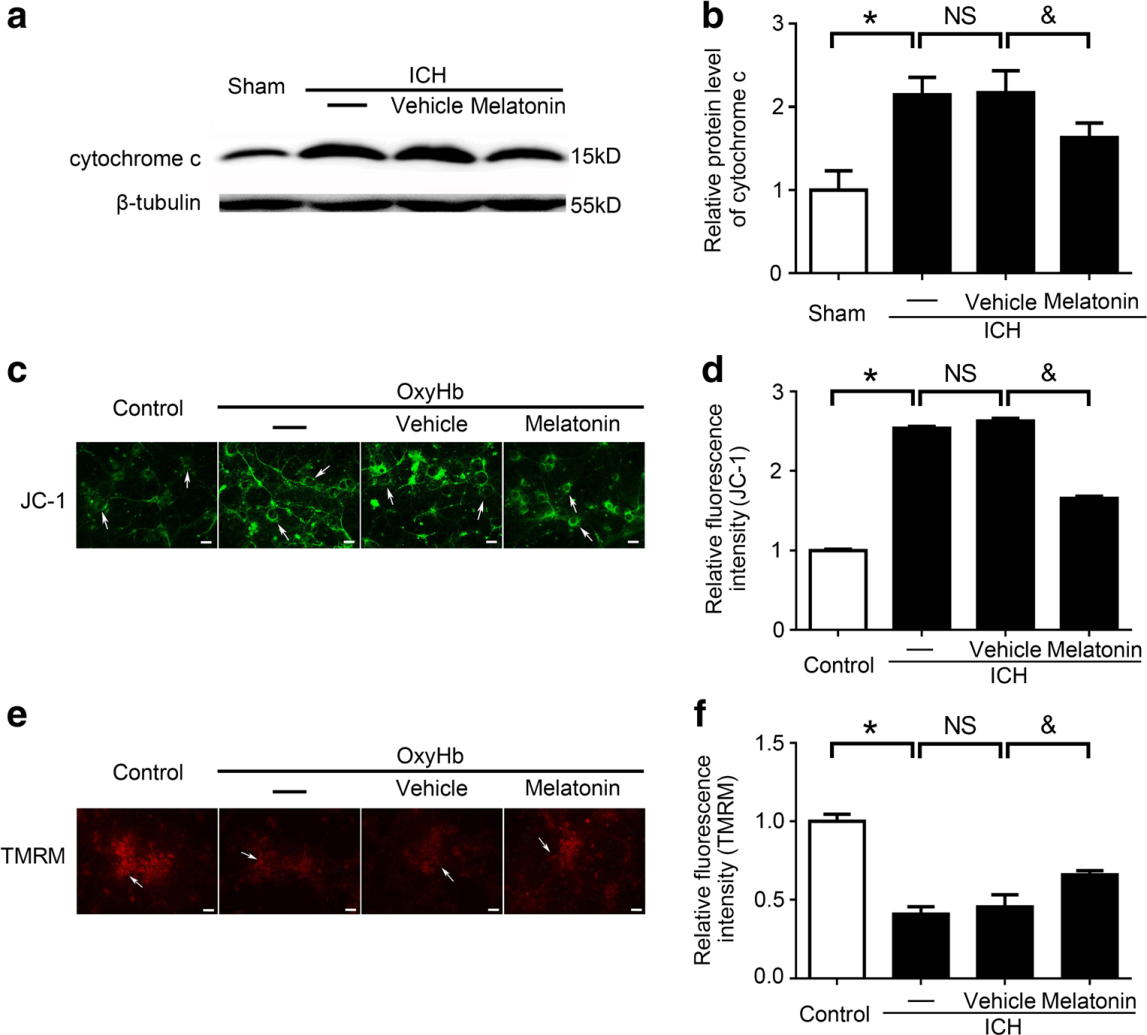
Melatonin suppressed mitochondrial damage indicator levels in brain tissues at 72 h after ICH and mitochondrial permeability transition pore (MPTP) opening in vitro. **a** Western blot analysis showed cytochrome c expression level in the sham, ICH, ICH + vehicle, and ICH + melatonin groups in vivo. **b** Relative cytochrome c levels were calculated based on densitometry analysis. The mean cytochrome c level of the sham group was normalized to 1.0. All data are displayed as a mean ± SEM, with *P < 0.05 and ^&^ *P* < 0.05 considered significant. *NS*, no significant difference, *n* = 6. The primary cultured neurons were stained by JC-1 (**c**) and TMRM (**e**) after OxyHb treatment and were then observed using fluorescence microscopy. *Arrows* indicate neurons; *scale bar* = 32 μm. The relative fluorescence intensity of JC-1 (**d**) and TMRM (**f**) was calculated using ImageJ. The mean fluorescence intensity values in the control group were normalized to 1.0. All data are displayed as mean ± SEM, with *P < 0.05 and ^&^ *P* < 0.05 deemed as significant difference; *NS*, no significant difference, *n* = 6

To define the effects of melatonin treatment in mitochondrial damage in brain tissues after ICH, the levels of cytochrome c in the cytoplasm, an indicator of mitochondrial damage, were detected by western blot analysis. The results demonstrated that, compared with the sham group, the levels of cytochrome c in the cytoplasm were obviously increased in the ICH group. However, melatonin reduced the levels of cytochrome c in the cytoplasm compared to the ICH + vehicle groups (Fig. 11a, b). These results suggested that melatonin plays an important role in protecting mitochondria injury after ICH in vivo.

JC-1 and TMRM are ideal fluorescent probes widely used to detect mitochondrial membrane potential (△Ψm). The mitochondrial transmembrane potential makes some lipophilic cationic fluorescent dyes such as JC-1 and TMRM to bind to the mitochondrial matrix, and the enhancement or decrease of fluorescence indicates the increase or decrease of electrical negativity of the mitochondrial inner membrane. MPTP opening was evaluated by observing the relative fluorescence intensity of JC-1 (Fig. 11c, d) and TMRM (Fig. 11e, f) in neurons in vitro. MPTP opening increased significantly in the OxyHb group compared to the sham group, while it decreased in the OxyHb + melatonin group compared with the OxyHb + vehicle group. These results indicated that melatonin can offer mitochondrial protection by decreasing OxyHb-induced MPTP opening in neurons in vitro.

## Discussion

The mechanisms contributing to SBI after ICH are complex and mainly attributed to the following: mechanical rupture of nerve and glial cells due to the space-occupying effect of the hematoma, excitatory amino acid toxicity, free radical induced destruction of intracranial cells via increased levels of ROS and other small molecules, inflammation induced by intravascular inflammatory cells (neutrophils, macrophages, and so on) and activated microglia in brain tissues, and apoptosis induced by a variety of mechanisms [6, 30–32].

Brain edema is a major and severe pathological event induced by ICH, with the mechanisms contributing to its formation being quite complex [33, 34]. The pathological process of ICH-induced brain edema mainly included the following: the space-occupying effects of the hematoma, the hydrostatic pressure generated during blood clot formation and retraction, destruction of the BBB, a coagulation cascade and the formation of thrombin, the dissolution of red blood cells and the toxic effect of hemoglobin, and secondary cerebral ischemia/reperfusion injury [35–39]. In this study, we found that melatonin significantly reduced ICH-induced brain edema. Furthermore, melatonin reduced the albumin concentration in the ICH group, thus indicating that melatonin can rehabilitate the BBB integrity. This rehabilitation of the BBB integrity may be an important contributing factor to the reduction of brain edema following melatonin treatment.

**Fig. 12:**
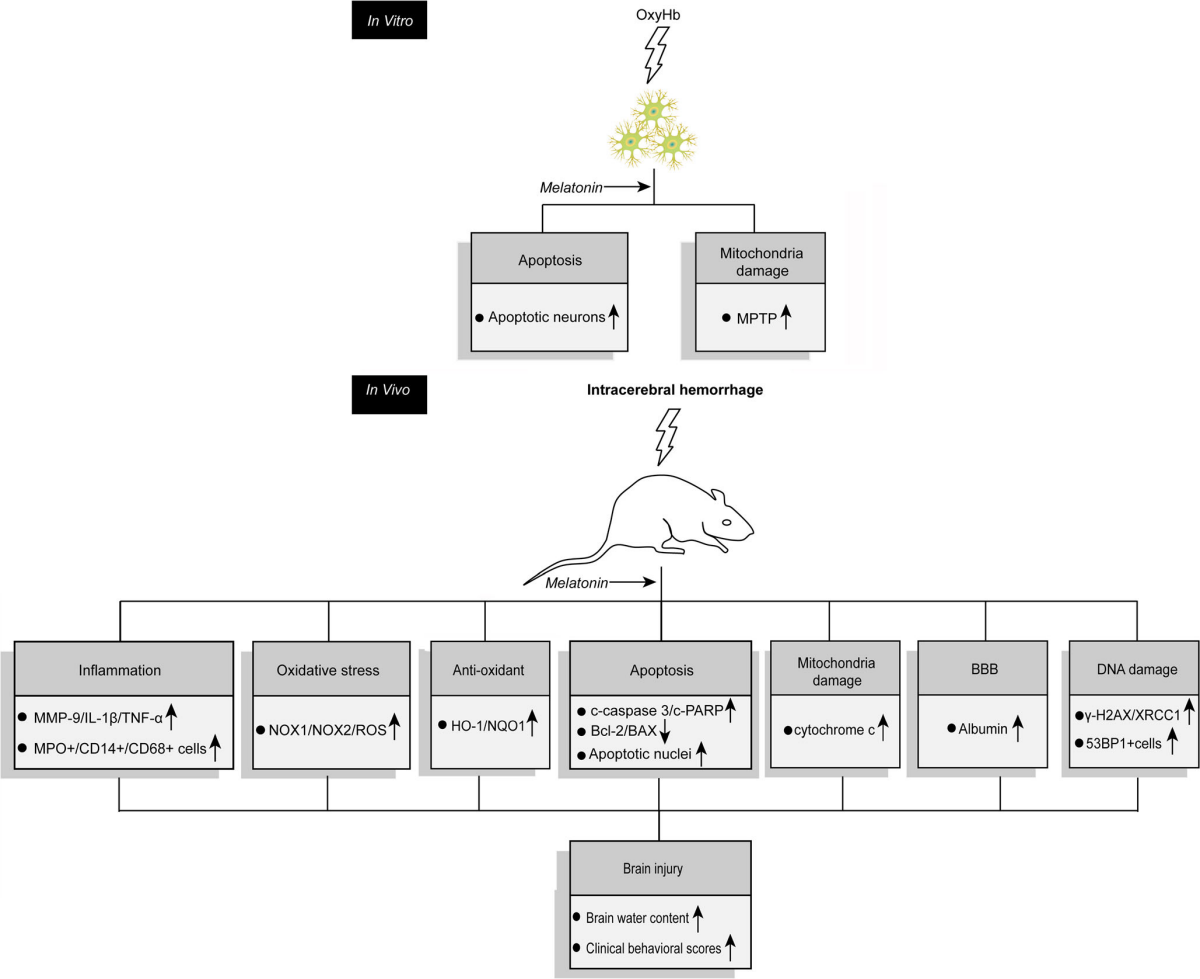
Proposed mechanisms underlying the positive therapeutic effects of melatonin treatment on SBI induced by ICH. *c-caspase 3* cleaved caspase 3, *c-PARP* cleaved poly(ADP-ribose) polymerase (PARP), *NOX* NADPH oxidase, *TNF* tumor necrotic factor, *MMP-9* matrix metalloproteinase 9, *IL-1β* interleukin-1β, *MPO* myeloperoxidase, *ROS* reactive oxygen species, *HO* heme oxygenase, *NQO1* NAD(P)H quinone oxidoreductase 1, *XRCC* X-ray repair complementing defective repair in Chinese hamster cells, *MPO* myeloperoxidase, *53BP1* p53-binding proteins 1

During the inflammatory reaction in brain tissues after ICH, the most basic signs are the activation of microglia and the infiltration of inflammatory cells [40]. Previous studies found that white blood cells and macrophages released from the hematoma infiltrated and blocked microblood vessels thereby reducing cerebral perfusion and damaging the BBB [41, 42]. TNF-α, IL-1β, and other cytotoxic molecules caused damage to neurons [43] and were involved in the process of SBI [44]. Previous studies showed that ischemia and hypoxia occurred in the brain tissues around the hematoma after ICH, which activated microglia, inflammatory cells, vascular endothelial cells, and so on, and then led to increased expression of MMP-9. MMP-9 caused vascular matrix degradation and disrupted BBB, thus contributing to brain edema [45–50]. Our study showed that melatonin can decrease inflammatory cytokine (TNF-α and IL-1β) levels and reduce inflammatory cell infiltration in brain tissues, thereby alleviating the inflammatory reaction after ICH (Fig. 12).

Oxidative stress played an essential role in the occurrence of ICH-induced brain edema and SBI. Following ICH induction, a large number of free radicals were released, and ROS led to membrane lipid peroxidation and protein and DNA oxidative damage [51–53]. HO-1 protected cells and tissues during oxidative stress as an important member of antioxidant [54–56]. NQO1 used NADH or NADPH as electron donors to catalyze the reduction of chinone compounds, thereby avoiding the formation of unstable semiquinone compounds [56, 57]. In this study, we found that melatonin increased the expressions of HO-1 and NQO1 after ICH, and these results indicated that melatonin can promote antioxidant in brain tissues after ICH. The major function of the NOX family was the production of ROS [58, 59]. Following ICH induction, the expression of NOX-1 and NOX-2 were upregulated and contributed to brain injury. Our study showed that increasing of NOX-1 and NOX-2 after ICH could be ameliorated by melatonin. Furthermore, melatonin decreased ROS levels in brain tissues after ICH, thereby alleviating oxidative stress.

Apoptosis was the main mechanism of early tissue injury in the region surrounding the hematoma after ICH. There were many factors which induced cell apoptosis after ICH, such as free radical cascade reaction, inflammation, cytokine stimulation, and the induction of thrombin and blood components. There were also various genes that regulated neuronal apoptosis, including BAX, which promoted apoptosis, and Bcl-2, which inhibited apoptosis [60, 61]. When the expression of Bcl-2 increased, the heterodimer Bcl-2-Bax formed and inhibited cell apoptosis. On the contrary, when the expression of Bax increased, the form of homodimer Bax/Bax was also increased and promoted cell apoptosis. The ratio of Bcl-2/Bax can reflect whether the cells tend to undergo apoptosis or survive after stimulation. When the level of Bcl-2 was increased while Bax was decreased, the ratio of Bcl-2/Bax increased, and cells tended to survive [62, 63]. In this study, we found that the ratio of Bcl-2/Bax in brain tissues decreased post-ICH but this decrease was alleviated by melatonin. At the start of cellular apoptosis, PARP was cleaved into two fragments by caspase 3, thereby inactivating PARP and leading to apoptosis [64, 65]. Neurons in the hippocampal CA1 region were closely related to learning and memory function of human and mammal and sensitive to ischemia. In recent years, researchers found that secondary cerebral ischemia probably occurred in the hippocampal CA1 region post-ICH. The delayed death of neurons in the hippocampal CA1 region after transient cerebral ischemia was the process of apoptosis [66, 67]. This study showed that melatonin can reduce apoptotic cell numbers around the hematoma in brain tissues after ICH.

The mitochondria, a eukaryotic organelle, consists of a bilayer that aids in ATP production, and is the site of intracellular oxygen free radical production and the main target of oxygen free radicals [68, 69]. Mitochondrial dysfunction can lead to a variety of intracellular signaling cascades, oxidative stress, and apoptosis, which play a crucial role in the progress of almost all diseases [70]. There was evidence that mitochondrial damage induced by cerebral I/R injury was directly related with neuronal apoptosis [71–73]. The mitochondria also have other important functions, such as ROS production, regulating cellular redox potentials and signal transduction, and controlling cellular apoptosis and gene expression [73, 74]. Following ICH induction, mitochondrial injury occurred as the result of MPTP opening; a variety of proteins including cytochrome c were released into the cytoplasm, being an important event that led to cell apoptosis [75, 76]. Melatonin reduced mitochondrial dysfunction by upregulating antioxidants, thus inhibiting MPTP opening. This study suggested that melatonin treatment significantly inhibited apoptosis and mitochondrial damage, with these findings substantiated both in vitro and in vivo. Additionally, ICH-induced DNA damage was also an important cause of SBI. When DNA was broken, 53BP1 and γ-H2AX were synthesized and released, with γ-H2AX providing an index of DNA damage and 53BP1 promoting DNA repair [77–79]. This study showed that melatonin treatment can alleviate ICH-induced DNA damage.

We also noticed that there are three papers from a group reporting that melatonin reduced oxidative stress, and provided brain protection after ICH, but it did not change the extent of brain edema or neurologic deficits in short-term outcomes [20, 80, 81]. These differences may due to the methods of induction of ICH (we used autologous whole blood, but they used bacterial collagenase in induction of ICH in rats). In collagenase ICH model, bacterial collagenase induced excessive inflammation response in the brain tissues; this should not be ignored in neuroprotection-related studies. In this study, we researched the neuroprotective effects of melatonin in autologous blood injection ICH model for the first time. Taking these researches together, using multiple models may be more appropriate in the study of brain injury following ICH.

One of the limitations of the present study is that it only covered a short period of time; thus, the long-term effects of melatonin on ICH remain unclear. Furthermore, only the effects of melatonin on inflammation, oxidative stress, apoptosis, and other brain injury were examined, but the mechanisms were not characterized. The relationship between inflammation, oxidative stress, and apoptosis in ICH thus remains unclear. Moreover, the direct effect of melatonin on the inflammatory reaction was not examined herein, although the reduction in inflammatory response was possible due to the reduction in the oxidative stress response. According to previous reports, receptors of melatonin are as follows: MT1 (on cell membrane), MT2 (on cell membrane), melatonin receptor type 1c, quinone reductase 2 enzyme (MT3 receptor, a detoxification enzyme), retinoid-related orphan nuclear hormone receptor, and GPR50 (X-linked melatonin-related orphan receptor) [82]. To date, there were only a few studies reporting that MT1 and MT2 receptors were expressed in the brain tissues in normal SD rats; however, whether expression levels of these two receptors were changed and other receptors of melatonin were expressed in brain tissues in rats after ICH have not been reported [83]. So it is necessary to make further investigations to identify whether the effects of melatonin in brain injury after ICH in rats were mediated by MT1 receptor, MT2 receptor, and (or) other receptors. Meanwhile, some researchers thought that melatonin maybe a pleiotropic agent that is capable of interfering with oxidative stress, cell apoptosis, inflammation, and so on, all of which would be useful in treating common pathological events taking place in ICH and other disease [84]. Thus, the main purpose of this study was investigating the effects of melatonin in secondary brain injury after ICH. Of course, the major mechanisms and the key targets of melatonin involved in melatonin-induced neuroprotective effects showed in this study would be explored in our future work.

In conclusion, we found that melatonin treatment can alleviate SBI and protect brain tissues after ICH by impacting apoptosis, inflammation, oxidative stress, DNA damage, brain edema, and BBB damage and reducing mitochondrial membrane permeability transition pore opening. This study indicated that melatonin may become an important treatment against mitochondrial dysfunction and ICH-induced disabilities.
